# Technology-Based Parenting and Digital Media Use: Adolescents’ Health in a Large, Cross-Sectional Study in Northern Italy

**DOI:** 10.3390/bs16030439

**Published:** 2026-03-17

**Authors:** Verena Barbieri, Giuliano Piccoliori, Adolf Engl, Doris Hager von Strobele-Prainsack, Christian Josef Wiedermann

**Affiliations:** Institute of General Practice and Public Health, Claudiana College of Health Professions, 39100 Bolzano, BZ, Italy

**Keywords:** children and adolescents, age, technology-based parenting, hours of digital media use, mental health, digital technologies

## Abstract

Background: Extended digital media consumption affects the mental and overall health of children and adolescents. The role of technology-based parenting (TBP) in limiting or controlling digital media use in this context is controversial. Methods: A representative sample of 5832 parents of schoolchildren aged 6 to 17 participated in an anonymous online survey in 2025. Correlation analysis identified simple associations with health-related parameters, and ANOVA models examined the relationship between TBP, digital media use and health-related parameters across children, early adolescents and late adolescents. Results: Digital media use increased with age, whereas TBP peaked at 11 years of age. In children, both variables were positively associated, but for late adolescents, the association was negative. For early adolescents, both factors were related to mental health symptoms. In late adolescents, both factors are related to sleep duration and physical activity. Social support was positively associated with TBP in early adolescents and negatively associated with digital media use in children and late adolescents. ANOVA showed that late adolescents using digital media between 2.5 and 3.5 h a day slept more when controlled by TBP. Conclusion: Children should limit their digital media use. Early adolescents need strong child–parent relationships. Late adolescents can achieve a healthier lifestyle with TBP. Age-specific information campaigns and intervention programs can support families in managing digital media use and promoting well-being.

## 1. Introduction

Digital media are deeply integrated into the daily routines of children and adolescents. Consequently, many families adopt technology-based parenting (TBP) strategies, utilizing tools such as Apple Family Sharing, Google Family Link, and third-party applications, to filter content or limit screen time and to regulate young people’s digital engagement.

Research indicates that setting limits and monitoring screen use are associated with reduced screen time and fewer problematic behaviors. However, punitive or overly surveillance-oriented approaches tend to correlate with poorer psychosocial outcomes, including diminished self-esteem and increased problematic behaviors (Nagata et al., 2025a; Hampton & Shin, 2023; Chen et al., 2025). Systematic reviews suggest that technical controls—such as filters, time limits, and monitoring tools—can decrease online risks and sometimes reduce overall screen time. Nonetheless, their positive impact on broader well-being is limited unless integrated within supportive parenting practices (Stoilova et al., 2020; Livingstone & Helsper, 2008; Valkenburg et al., 2013).

Several established theories help structure the dynamics of TBP. “Parental Mediation Theory” categorizes strategies into restrictive (rules, limits, filters), active/enabling (discussion, guidance), and co-use approaches. The effectiveness of these strategies depends on how they are integrated into daily parenting routines (Livingstone & Helsper, 2008; Valkenburg et al., 2013). Additionally, “Ecological Systems Theory” situates family practices within broader social and resource contexts—such as parental education, family structure, migration background—that can either enable or hinder effective implementation of TBP (Bronfenbrenner, 1979). “Social Cognitive Theory (Reciprocal Determinism)” underscores the mutual influence between child behavior, parenting and environmental factors over time (Bandura, 1986). This interconnectedness complicates causal inferences in cross-sectional data and makes age-specific analyses essential.

Converging evidence presents a nuanced view of TBP as a “double-edged” approach. Developmentally, adolescents’ increasing need for autonomy means that transparent, collaborative controls can be beneficial, whereas punitive or opaque measures may be counterproductive (Stoilova et al., 2020). In terms of health behaviors, bedtime device rules are consistently linked to longer sleep duration and improved daytime functioning (Mammeri et al., 2025). The family environment moderates these effects: warmth, support, and collaborative rule-setting enhance the benefits, whereas stress and limited resources diminish them.

Thus, TBP employs strategies for parents to understand, monitor, and manage their children’s engagement with digital technology in a broader social and possibly age-dependent context. Our study aims to address these issues in relation to age by defining TBP as the use of any digital tool to manage young people’s device use. We use social support as a parameter for social and familial dealing with daily routines, and socioeconomic parameters regard contexts of the ecological system theory. Finally, lifestyle parameters such as hours of daily sleep are analyzed in this context to understand their associations with health behaviors.

Beyond parent–child interactions, research over the past two decades has linked screen exposure to various outcomes, including sleep patterns, psychosocial health, and lifestyle behaviors (Vandewater et al., 2005; Ramirez et al., 2011). These findings remain heterogeneous and vary by age (Sanders et al., 2016) and context (Wartella et al., 2013). In early adolescence, less screen time has been linked to problematic Internet and social media use, mental health problems and problematic parent–child relationships (Nagata et al., 2025b; Al-Shoaibi et al., 2024; Nagata et al., 2024; Byun et al., 2024; Jörren et al., 2023). Conversely, in late adolescence (Ngantcha et al., 2018; Surís et al., 2023), spending more than two hours daily on screens is linked to lower life satisfaction, decreased physical activity, school bullying, and grade repetition.

Overall, these insights raise questions about whether TBP functions primarily as a preventive, adaptive strategy promoting healthier habits—such as earlier bedtimes, lower BMI, fewer mental health problems and fewer problems in child–parent relationships (Priftis & Panagiotakos, 2023)—or as a reactive, authoritarian response to excessive screen and social media use (Francis et al., 2021; Sampasa-Kanyinga et al., 2020).

Variations across age groups underscore the need for age-dependent research examining how TBP interacts with screen time use and how both factors relate to family, health and sociodemographic variables.

Addressing this knowledge gap, the current study analyses a large, cross-sectional, age-specific, population-based sample collected post-pandemic and focused on contemporary TBP practices and hours of digital media use.

This study tests the following hypotheses:

**H1.**  *Across developmental stages (6–9, 10–13, and 14–17 years)—TBP use (presence vs. absence) is differently associated with patterns of digital media use (school-related and discretionary).*

**H2.**  *Family, demographic, lifestyle and health-related factors co-occur differently with TBP and digital media use, indicating that simply reducing screen time may not suffice to improve children’s health.*

**H3.**  *The interaction between TBP and hours of screen time use may be associated with health-relevant outcomes, including sleep duration, psychosocial difficulties, psychosomatic complaints, physical activity, weight status, perceived health, and school-related stress.*

This age-specific, literature-informed approach seeks to describe when TBP is related to more favorable health-related patterns and when it may indicate existing difficulties. The data provide insights into the daily lives and challenges faced by youths in the post-pandemic era. Ultimately, the goal is to generate actionable evidence for clinicians, educators, and families to support healthier digital media practices.

## 2. Materials and Methods

### 2.1. Study Design and Sample

The Corona and Psyche—South Tyrol (COP-S) survey employed a repeated cross-sectional design. The present study analyzed data from the fourth wave, conducted in 2025. This research aimed to evaluate the mental-health- and health-related factors among children and adolescents in South Tyrol, focusing on schoolchildren aged 6 to 19 years. The primary goal was to provide a current, representative snapshot of mental health trends and lifestyle behaviors among this demographic. Additionally, this study sought to contribute to a broader understanding of the actual health status and digital media use after the pandemic.

The methodology involved an anonymous online survey targeting parents of schoolchildren across South Tyrol. The survey was conducted from 17 March to 13 April 2025, via the SoSci Survey platform (Version 3.2.46 SoSci Survey GmbH, Munich, Germany).

The present study presents parent (proxy)-reported data, recruited from all schools across the province by contacting the parents via email and providing a link to an online questionnaire. Parents provided informed consent, and adolescents aged 11 years and older also consented to participate.

The questionnaire was distributed to over 40,000 families. After the first invitation, a reminder was sent to all families after two weeks. A total of 9745 (response rate of approximately 23%) parents responded to the study, and approximately 80% of the responses were suitable for analysis after data cleaning. The analyses considered datasets from schoolchildren aged 6 to 17 years, containing information on the use of TBP. The final sample comprised 5832 participants.

The mean age of the children was 11.15 years; 50.9% were male, and 10.0% had a single parent. The sample’s demographic distribution, including age, gender, and family structure, closely matched regional statistics from ASTAT, the provincial institute of statistics in South Tyrol, ensuring representativeness.

### 2.2. Main Outcomes: Technology-Based Parenting and Hours of Digital Media Use

The use of TBP was assessed using a single question asking whether parents used settings like Family Link, Apple Family Sharing, or Time Limit, with response options of “yes” or “no”.

Participants were asked to provide information on the number of hours spent on digital media per day and were evaluated separately for private and educational contexts. This was done through the question “How many hours does your child spend in total per day with computer, smartphone, tablet, console… (i.e., digital devices)? for private/school concerns?” Responses were rated on a 7-point Likert scale from 1 = “never” to 7 = “five hours or more”. This detailed assessment aimed to evaluate the extent of digital media engagement among young people, considering both leisure and educational activities.

### 2.3. Sociodemographic and Lifestyle Measures

The variables included children’s age and gender, family structure (single parenthood), migration background, and parental educational attainment, as measured by the Comparative Analysis of Social Mobility in Industrial Nations (CASMIN) index (Brauns et al., 2003).

Adolescents were categorized into early adolescents (10–13 years) and late adolescents (14–17 years), based on the rationale that balanced existing definitions and ensured comparable group sizes. Children aged 6 to 9 years were analyzed separately. Physical activity frequency was recorded as the number of days per week with more than one hour of activity, scored from 1 (0 days) to 8 (7 days). School-related stress was measured using a four-point Likert scale from 1 = “not at all” to 4 = “very much”. Parental assistance with schoolwork in the actual school year was similarly assessed using a four-point Likert scale ranging from 1 = “never” to 7 = “always” and with the option 6 = “not applicable”. Sleep duration was calculated from typical bedtimes and wake-up times on school days, providing an estimate of total sleep hours. Overall health status was rated on a five-point Likert scale ranging from “excellent” to “bad”, with the scale inverted for analysis purposes.

Social support was evaluated using the Multidimensional Scale of Perceived Social Support (MSPSS), a 12-item instrument that measures perceived support from family, friends, and others. Responses ranged from 1 (“strongly disagree”) to 7 (“strongly agree”), with higher scores indicating greater perceived support (Zimet, 2016).

Socioeconomic status was assessed using the Family Affluence Scale (FAS III), which includes six items related to material wealth indicators such as ownership of a computer, car, own bedroom, bathroom, dishwasher, and frequency of vacations. The total score ranges from 0 to 13, reflecting family material well-being (Hobza et al., 2017; Currie et al., 2024; Hartley et al., 2016; Corell et al., 2021).

Anthropometric data, specifically height and weight, were collected to calculate the body mass index (BMI).

The Health Behavior in School-aged Children Symptom Checklist (HBSC-SCL) identified psychosomatic complaints. The presence of eight psychosomatic symptoms—headaches, stomach-aches, backaches, feeling down, irritability, feeling nervous, sleep problems, and dizziness—over the past week was assessed. Responses were recorded on a five-point scale ranging from 1 = “daily” to 5 = “not at all” (Haugland et al., 2001; Heinz et al., 2022). The number of different complaints per week was used as a count variable, ranging from 0 to 8.

Adolescents’ mental health was evaluated using the Strengths and Difficulties Questionnaire (SDQ), which measures emotional symptoms, conduct problems, hyperactivity/inattention, peer relationship problems, and prosocial behavior. The total problem score, derived from the first four subscales, ranged from 0 to 40, with higher scores indicating more difficulties. Lower prosocial behavior scores signified greater problems. The SDQ demonstrated good internal consistency, with Cronbach’s alpha values of 0.82 (German version) and 0.81 (Italian version) (Goodman, 2001; Tobia & Marzocchi, 2018).

Self-assessed parental health literacy was measured using the HLS-EU-Q16 questionnaire (Sørensen et al., 2013; Lorini et al., 2019; Lorini et al., 2017; Tiller et al., 2015). The total score ranged from 0 to 16, with higher values indicating better health literacy. The instrument showed high reliability, with Cronbach’s alpha values of 0.799 for the Italian version and 0.88 for the German version (Lorini et al., 2019; Tiller et al., 2015).

### 2.4. Data Analysis

Data analysis involved descriptive statistics, with means (Ms) and standard deviations (SDs) for metric variables and counts and percentages for categorical variables. Group differences were tested using chi-square tests for nominal data and Mann–Whitney U tests for ordinal and continuous variables. Correlations were examined using Phi coefficients for nominal data, point-biserial coefficients for continuous–dichotomous pairs, and Pearson’s correlation for continuous variables. The reliability of composite scores was evaluated using Cronbach’s alpha. To explore relationships among variables, univariate two-factorial analysis of variance (ANOVA) models were employed, with Levene’s test assessing homogeneity of variances. Graphical visualization of marginal means facilitated interpretation, and post hoc comparisons used the Bonferroni correction. Effect sizes were expressed as partial eta squared, interpreted as small (0.01), medium (0.06), and large (0.14). A minimum sample size of 357 cases was calculated to ensure adequate power for the ANOVA models, considering eight groups, a medium effect size (0.25), an alpha of 0.05, and a power of 95%. All statistical procedures were conducted using IBM SPSS Statistics for Windows (version 25.0; IBM Corp., Armonk, NY, USA).

## 3. Results

Of the participating schoolchildren, 2966 (50.9%) were male, 2865 (49.1%) were female, and one was identified as diverse. This single case was not considered in gender-specific analyses. Approximately 16% of the participants reported a low CASMIN status. Urban residency was reported by 30.5% of the participants, with 10% living in single-parent households, 11.5% having a migration background, and 89.2% of parents being female. Psychometric reliability was high, with Cronbach’s alpha values of 0.983 for MSPSS, 0.838 for SDQ, and 0.851 for HLS-EU-Q16, indicating strong internal consistency.

### 3.1. Technology-Based Parenting and Hours of Digital Media Use

**Figure 1:**
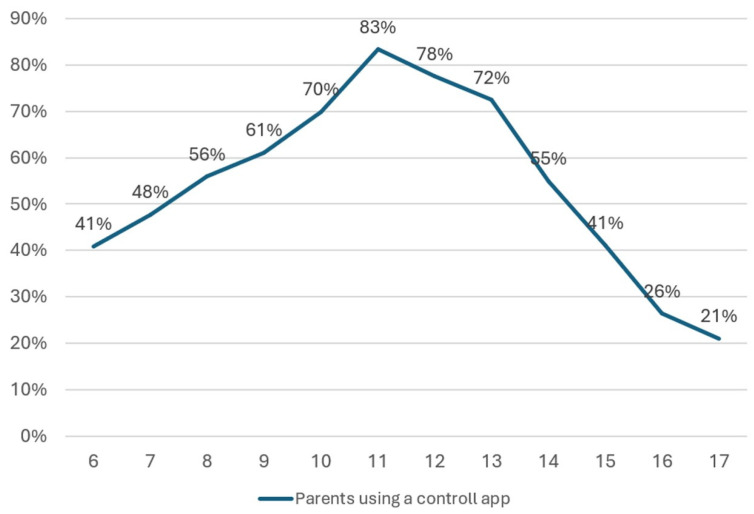
Percentage of use of technology-based parenting (y-axis) for children and adolescents aged 6 to 17 years (x-axis).

For children and adolescents aged 6–17 years, the overall rate of parents using TBP was 54.9%. This rate varied with age, as shown in Figure 1.

**Figure 2:**
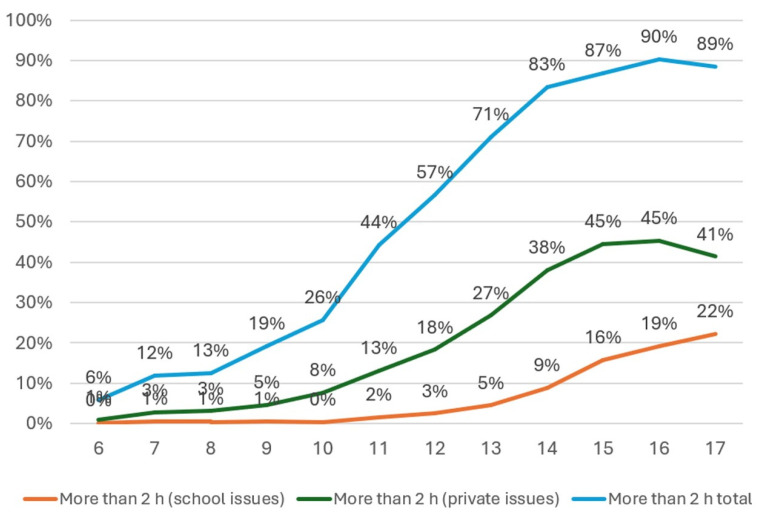
Percentage of extended use of digital media (y-axis) for children aged 6–17 years (x-axis).

Notably, 5.7% of parents reported their children used digital media for more than two hours daily for academic purposes, while 18.9% exceeded this duration for personal reasons. Combining these figures, nearly half (46.5%) of children used digital media for over two hours daily. Age-specific data revealed that TBP use increased with age, peaking at 75.5% among 10–13-year-olds and then declining to 36.7% in the 14–17 group. Correspondingly, the proportion exceeding two hours of digital media use was 12.4% in 6–9-year-olds, 48.7% in 10–13-year-olds, and a striking 87.1% in adolescents aged 14–17 years. Usage for school purposes was minimal in younger children but increased significantly in older adolescents, reaching 16.2%, while private use was more prevalent, especially in the oldest group, at 42.2%. Detailed age-specific data are presented in Figure 2.

**Figure 3:**
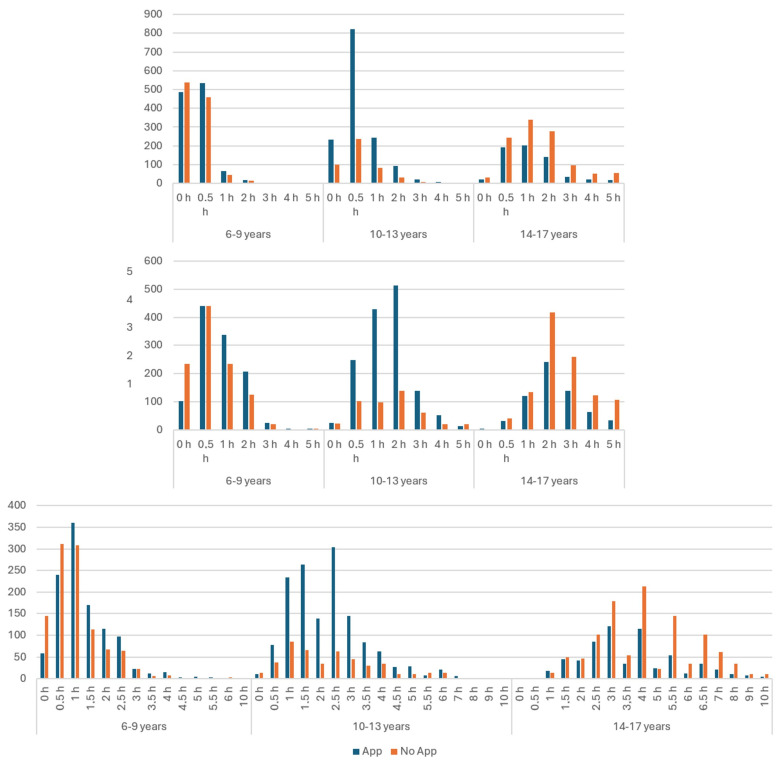
Digital media use in absolute numbers for children controlled by TBP and children not controlled by TBP for school issues (**upper** panel), private issues (**middle** panel) and the sum of both (**lower** panel).

Table 1 and Figure 3 illustrates the hours spent on digital media for school and private purposes, as well as the total use, for children with and without TBP control across age groups. In children aged 6–9 years, TBP was positively correlated with hours of media use for school, both private and total. For ages 10–13, no correlations were observed for total or for school purposes, but a small negative correlation was found for private use. In adolescents aged 14–17, negative correlations emerged across all measures: school, private and total.

### 3.2. Associations with Demographic, Lifestyle and Health-Related Factors

Table 2 summarizes the relationship between demographic, lifestyle and health-related parameters with TBP use and with total digital media use per age group.

Across all age groups, TBP use was not associated with gender, SDQ emotional score, SDQ peer score, number of psychosomatic complaints, general health state or school stress.

In children aged 6 to 9 years, the use of TBP was positively linked to a low CASMIN index, higher FAS III, a higher SDQ prosocial score (indicating better prosocial behavior) and a higher BMI. These findings suggest that TBP in childhood is mainly associated with sociocultural factors and sparsely with health factors.

For children aged 10–13 years, TBP use was negatively associated with migration background, single parenthood and a low CASMIN index. Conversely, it was positively associated with higher MSPSS scores, a higher SDQ total score, a higher SDQ conduct score and a higher SDQ hyperactivity score, indicating that better social support and poorer mental health are linked to increased TBP use.

Among adolescents aged 14–17 years, TBP was positively related to urban residence, hours of sleep, higher parental health literacy, lower BMI, hours of sports, and more parental help with school problems, reflecting a trend toward healthier lifestyles with TBP engagement.

Regarding digital media hours, no relationship with urban residency was observed across age groups. In all age groups, a negative association was observed with a higher SDQ prosocial score, more hours of sleep and hours of physical activity, and a higher general health state. Positive associations were found with higher SDQ total scores, higher SDQ emotional scores, higher SDQ conduct scores, higher SDQ hyperactivity scores, higher BMI, more psychosomatic complaints and higher school stress.

For children aged 6 to 9, higher digital media use was positively related to migration background, single parenthood, low CASMIN index, and more parental help with schoolwork. A negative association was found with female gender, MSPSS scores and higher parental health literacy.

For early adolescents aged 10–13 years, use of digital media was related positively to migration background, single parenthood and a low CASMIN index.

For adolescents aged 14–17, digital media use was positively related to female gender and higher FAS III. A negative relationship was found with a low CASMIN index.

Only a few parameters were linked to both TBP and digital media use. In children aged 6 to 9 years, these included a low CASMIN index, BMI and SDQ prosocial score. For ages 10–13, the associations involved migration background, single parenthood and a low CASMIN index, SDQ total score, SDQ conduct score and SDQ hyperactivity score. For adolescents aged 14–17 years, relevant factors included hours of sleep, BMI and hours of sports.

Overall, the findings suggest that sociocultural factors are associated with TBP or screen time in childhood, whereas associations with both variables were scarce. Some mental health parameters and sociocultural factors were associated with both variables in early adolescence. Lifestyle factors were associated with both variables in late adolescence. The interactions between these variables were further examined through two-way ANOVA analyses, which assess the effects of TBP and digital media use on health and lifestyle outcomes across different age groups.

### 3.3. Two-Way ANOVA for Health-Related and Lifestyle Parameters

We employed a two-way ANOVA to examine the effects of TBP and digital media use on various health and lifestyle outcomes across different age groups. Digital media use was categorized into four groups based on hours per day: 0–0.5 h, 1–2 h, 2.5–3.5 h, and 4 or more hours. Table 3 presents absolute count data and percentages within each age group.

BMI and SDQ prosocial score were analyzed in the youngest age group (6 to 9). Due to different group counts and significant Levene’s test results, indicating variance differences across groups, the results were not interpreted and are not presented. Similarly, physical activity in the 14–17 age group was not analyzed because of the same issues.

**Figure 4:**
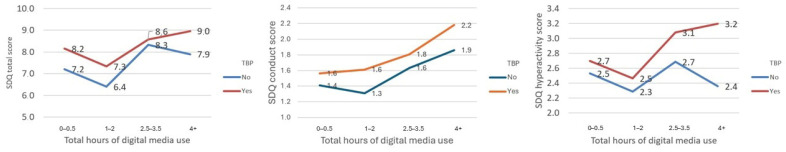
Marginal mean values derived from two-way ANOVA models examining the main and interaction effects of technology-based parenting (TBP; yes/no) and categories of daily digital media use. Outcomes for early adolescents aged 10–13 years (SDQ total score, SDQ hyperactivity, SDQ conduct problems).

**Figure 5:**
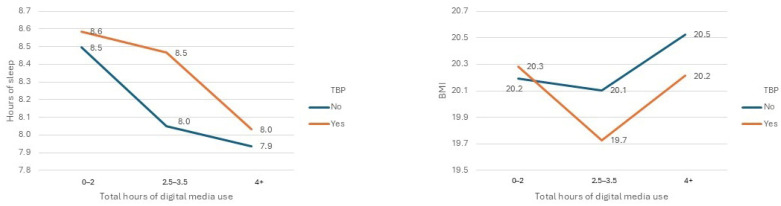
Outcomes for late adolescents aged 14–17 years (body mass index and hours of sleep). Digital media use categories correspond to those reported in Table 3.

For the middle age group (10 to 13), we analyzed the outcome variables SDQ total score, SDQ conduct score, and SDQ hyperactivity score. For late adolescents (14–17 years), the outcomes hours of sleep, BMI and hours of physical activity were analyzed. Table 4 presents marginal mean values with corresponding confidence intervals for ANOVA models with a non-significant Levene’s test; Figure 4 (10 to 13) and Figure 5 (14 to 17) shows the corresponding marginal distributions.

In the age group of 10–13 years, the ANOVA with the outcome variable total SDQ was significant (degrees of freedom (df = 7; F = 4.318; *p* < 0.001); both main effects, TBP (df = 1, F = 4.385; *p* = 0.036; effect size = 0.003) and hours of digital media use (df = 3; F = 6.954; *p* < 0.001; effect size = 0.012) were significant, while the interaction term (TBP × hours of digital media use) was not significant. Bonferroni-corrected post hoc tests revealed significant differences between the 1–2 h group and the 2.5–3.5 h group (*p* < 0.001), as well as the 1–2 h group and the 4+ h group (*p* = 0.006).

The overall ANOVA model analyzing the SDQ sub-score for hyperactivity was significant (df = 7; F = 4.66; *p* < 0.001); both main effects TBP (df = 1; F = 6.43; *p* = 0.011; effect size = 0.004) and number of hours for digital media use (df = 3; F = 3.83; *p* = 0.009; effect size = 0.007) were significant, and the interaction term was not significant. Bonferroni-corrected post hoc tests revealed a significant difference between the 1–2 h group and the group using digital media for 2.5–3.5 h (*p* = 0.001). The ANOVA model for the outcome SDQ conduct problems was significant (df = 7; F = 4.26; *p* < 0.001); both main effects, TBP (df = 1; F = 5.07; *p* = 0.024; effect size = 0.003) and hours of digital media use (df = 3; F = 6.64; *p* < 0.001; effect size = 0.011), were significant, while the interaction term was not significant. Bonferroni-corrected post hoc tests were significant for the groups using digital media for 0–0.5 h and 4+ h (*p* = 0.022) and the groups using digital media for 1–2 h and 4+ h (*p* < 0.001).

In the age group of 14–17 years, the ANOVA model for the outcome variable BMI was significant (df = 5; F = 2.63; *p* = 0.023; effect size = 0.005); the main effect of TBP was not significant, but the main effect hours of digital media use was significant (df = 2; F = 3.59; *p* = 0.028). The interaction term was not significant. Bonferroni-corrected post hoc tests revealed a significant difference between the group with 2.5–3.5 h of digital media use and that with 4+ h of digital media use (*p* = 0.023).

The ANOVA model for the outcome variable hours of sleep was significant (df = 5; F = 40.88; *p* < 0.001); the main effects TBP (df = 1; F = 19.85; *p* < 0.001, effect size = 0.012) and hours of digital media use (df = 2; F = 50.69; *p* < 0.001; effect size = 0.06) were significant. All subgroups of hours of use of digital media differed significantly (*p* < 0.001) from each other in post hoc tests, with less sleep associated with more hours. The interaction term TBP × hours of digital media use was significant (df = 2; F = 7.75; *p* < 0.001). Post hoc tests revealed a significant difference between TBP use, yes/no, in the 2.5–3.5 h group (*p* < 0.001; effect size = 0.025).

## 4. Discussion

We performed an in-depth examination of digital media consumption and its relationship with TBP across various developmental stages in children and adolescents. The study underscores the significance of age-specific patterns, illustrating how digital media engagement and TBP are interconnected with health, behavioral, and psychosocial outcomes. Our findings partly align with the results of Sanders et al. (2016), where TBP was negatively correlated with screen time in early childhood (3–7 years), marginally negative in middle childhood (8–12 years), and not significant in adolescence (13–17 years). Generally, excessive digital media use was related to less sleep and less physical activity, a higher BMI, more school stress and higher SDQ scores, as well as more psychosomatic complaints in all age groups. These new results, combined with the effects of other factors, can be interpreted within specific developmental contexts for each age group.

### 4.1. Children (6–9 Years)

The data indicated a positive correlation between TBP and digital media use, suggesting that TBP is used concurrently with digital media use. Notably, the associations of TBP with health-related or lifestyle variables were minimal in this age group. Small associations emerged for children from socioeconomically disadvantaged backgrounds. Prior research (Wartella et al., 2013) indicated that parents of younger children, who may be utilizing general adaptive parenting strategies, frequently report confusion or difficulties specific to the management of their child’s use of media devices at home.

However, socioeconomic factors significantly influence media behaviors, with children from disadvantaged backgrounds—such as those with single parents, low parental education, limited health literacy, or migration backgrounds—exhibiting higher digital media use. This highlights the sociocultural components that shape media engagement and emphasizes the need for targeted educational interventions. Families with low socioeconomic status could benefit from tailored information about the risks associated with high digital media consumption. Schools, teachers, and social institutions may represent relevant channels for disseminating such information. Although immediate health risks were not evident in this age group, early guidance on responsible digital media use may foster healthier habits and prevent potential issues later in development.

### 4.2. Early Adolescents (10 to 13 Years)

No significant correlation between the duration of digital media use and TBP was found. The absence of a clear link may be explained by the balancing act children perform between their desire for independence and parental regulation, as discussed by Francis et al. (2021). Parental perceptions often frame screen time as a conflict between children’s autonomy and parents’ efforts to impose limits. Higher TBP scores were associated with increased social support and elevated behavioral issues. Both very low and very high digital media engagement correlated with increased behavioral problems, forming a U-shaped relationship consistent with previous findings (Przybylski et al., 2020). This suggests that both excessive and minimal media use can be detrimental to mental health. Parental monitoring and establishing clear screen time boundaries are effective strategies for reducing problematic digital media use (Nagata et al., 2025b). Conversely, increased family conflict was linked to more screen time, emphasizing the importance of a supportive family environment (Al-Shoaibi et al., 2024). Elevated screen time was also linked to mental health issues such as depression, hyperactivity, and conduct problems, although these associations were modest (Nagata et al., 2024). Additionally, we found that children from vulnerable populations tended to have less parental oversight over media use and engaged more extensively with digital platforms. These findings highlight the relevance of considering the socioeconomic context when addressing digital media use and suggest that accessible information and strategies can promote healthier habits. A supportive family environment and social support networks are critical in managing TBP, given the established links between mental health and media use. Longitudinal studies, such as the German KIGGS and BELLA projects (Jörren et al., 2023), are necessary to clarify whether mental health issues lead to increased media consumption or vice versa. Such research can inform targeted interventions aimed at reducing mental health problems associated with parenting stress.

The findings suggest that it may be important to help families find strategies to manage screen time and not leave parents without support regarding this vulnerable age group. One important approach can be a school-based approach, teaching adolescents about healthy digital media use and providing alternatives to screen time. Such school-based interventions have the benefit of reaching families with low socioeconomic backgrounds. Additionally, schools can promote parent guidelines for the use of digital media and TBP.

### 4.3. Late Adolescents (14–17 Years)

A negative correlation was observed between TBP and more hours of digital media use. The use of TBP and reduced digital media use has been linked to lifestyle factors, particularly to more sleep. Thus, parental oversight may not only curtail screen time but also promote overall well-being. In 2018, adolescents reported spending approximately three hours daily on various media, such as television, video games, and computers (Ngantcha et al., 2018). Spending more than two hours on media was associated with lower life satisfaction, decreased physical activity, experiences of school bullying, and grade repetition. Socioeconomic status emerged as a key predictor of screen time use.

Supporting evidence from (Marciano & Camerini, 2021), found that less screen time (up to 2 h a day), sufficient sleep (8–10 h) and engagement in physical activity (moderate-to-vigorous physical activity [MVPA] at least 60 min/day) positively influence academic achievement in adolescence. These findings inform policymakers and educational institutions, underscoring the importance of balanced routines and resource support. The 24 h movement guidelines (Suchert et al., 2023; Sampasa-Kanyinga et al., 2022) provide practical frameworks for promoting healthy behaviors among youth and are suitable for dissemination among parents, teachers, and young people to prevent unhealthy lifestyle habits.

### 4.4. Implications for Interpretation and Future Research

The observed age-specific patterns indicate that the associations between TBP and digital media use are context-dependent and vary across developmental stages. These findings may help contextualize the heterogeneous results reported in prior studies and underscore the analytical value of age stratification in research on media-related parenting practices.

The current results should be viewed as descriptive and hypothesis-generating, with longitudinal research needed to clarify the causal relationships and reciprocal processes between parenting practices and digital media use, as well as to disentangle age-related patterns from cohort and contextual influences. Incorporating more nuanced measures of TBP and media contexts may further elucidate the underlying mechanisms influencing these behaviors.

### 4.5. Strengths and Limitations

This study provides up-to-date insights into the complex relationships between TBP and digital media use among children, early adolescents and late adolescents after the pandemic. Owing to the cross-sectional nature of this study, the results are limited to presenting associations. Further longitudinal research is needed to understand the effects of TBP use. The collected data present the schoolchild population of a region in Italy and may be applicable to other middle-European societies, but they cannot represent the general status of youth worldwide. Data can help to implement school-based interventions in the region, whereas other regions may meet other requirements.

This study investigated self-reported daily digital media use for school and private purposes. We did not differentiate according to the type of media use. Thus, we obtained indications about the extent; however, further investigations are needed to understand the effects of different types of digital media use on youths’ mental health.

Finally, we conducted an anonymous online survey with email invitations, which yielded a response rate of approximately 23%. The value and practicability of online child mental health surveys were discussed in (Goodman, 2013), showing that with a response rate of approximately 20%, results mostly replicate results from other studies. For pandemic times, the usability of online surveys is discussed in (Kumar et al., 2021), stating that with email invitation, a percentage of about 30% is reachable and is comparable to other surveys. Although the response rate was relatively low, the age and gender of the schoolchildren corresponded to official statistics as well as the percentage of single parents.

## 5. Conclusions

The results indicated that the relationship between TBP, digital media use, and health-related indicators differed across developmental stages, underscoring the relevance of age as an analytical dimension in research on digital media and parenting.

Possible interventions may involve minimizing digital media use in young children, promoting positive family dynamics in early adolescence, and employing TBP strategies in late adolescence to support overall health. Across age groups, higher levels of digital media use were consistently associated with less favorable lifestyles and psychosocial indicators. In contrast, TBP showed distinct age-related associations, appearing more closely linked to social and behavioral characteristics in early adolescence and lifestyle-related indicators in late adolescence. Taken together, these findings highlight the contextual nature of TBP and suggest that its role cannot be understood independently of age patterns.

The findings of (Theopilus et al., 2024), suggest that digital interventions should not only focus on restrictions but also on suggesting substitutive activities for children. Developing children’s competencies to combat addictive behaviors, improving digital literacy in children and parents, and supporting parental decision-making to promote healthy digital behaviors in their children is suggested. Society as a whole—comprising policymakers, educators, parents, and adolescents—must collaborate to develop effective strategies that balance digital engagement with healthy behaviors. Early education, school-based programs, and family support systems are essential components in cultivating responsible digital media use, ultimately fostering healthier developmental trajectories across all age groups.

## Figures and Tables

**Table 1:** Point-biserial coefficient for TBP (yes/no) with hours of digital media use per age group.

	6–9 Years	11–13 Years	14–17 Years
School purpose	0.062 **	−0.008	−0.110 ***
Private purpose	0.147 ***	−0.046 *	−0.108 ***
School and private purpose	0.145 ***	−0.040	−0.146 ***

Positive coefficients indicate higher media use among participants with TBP, whereas negative coefficients indicate lower media use. * *p* < 0.05; ** *p* < 0.01; *** *p* < 0.001.

**Table 2:** Relationships of TBP use and number of hours spent with digital media with demographic, lifestyle and health-related parameters per age group. (Phi coefficient for two nominal datapoints; point-biserial coefficient for one continuous and one dichotomous variable; Pearson’s correlation coefficient for two continuous variables).

	TBP			Number of Hours with Digital Media Total		
	6–9 Years	10–13 Years	14–17 Years	6–9 Years	10–13 Years	14–17 Years
Female gender child	−0.006	0.019	−0.012	−0.045 *	−0.039	0.077 **
Urban residence	0.003	−0.032	0.056 *	0.009	−0.031	0.037
Migration background	−0.021	−0.057 *	−0.010	0.057 **	0.046 *	0.030
Single parenthood	0.041	−0.057 *	−0.010	0.057 **	0.111 ***	0.021
Low CASMIN	0.063 **	−0.078 **	−0.021	0.056 **	0.096 ***	−0.059 *
FAS III	0.076 ***	0.031	0.031	0.014	−0.025	0.049 *
MSPSS	−0.027	0.063 *	0.029	−0.081 ***	−0.029	0.114 ***
MSPSS family	−0.030	0.067 *	0.029	−0.077 ***	−0.032	0.122 ***
MSPSS friends	−0.005	0.051 *	0.003	−0.057 **	−0.040	0.095 ***
MSPSS others	−0.029	0.064 *	0.036	−0.093 ***	−0.029	0.112 ***
Hours of sleep	−0.025	0.015	0.168 ***	−0.079 ***	−0.322 ***	−0.254 ***
SDQ total score	0.005	0.049 *	−0.007	0.097 ***	0.099 ***	0.170 ***
SDQ emotional score	−0.017	0.030	−0.020	0.072 **	0.064 *	0.181 ***
SDQ conduct score	−0.011	0.062 *	−0.014	0.049 *	0.119 ***	0.142 ***
SDQ hyperactivity score	0.034	0.060 *	0.021	0.059 **	0.094 ***	0.096 ***
SDQ peer score	−0.010	−0.031	−0.024	0.110 ***	0.042	0.081 **
SDQ prosocial score	0.057 *	0.020	0.023	−0.078 ***	−0.098 ***	−0.117 ***
Health literacy	0.037	0.036	0.063 *	−0.101	−0.041	−0.040
BMI	0.055 *	−0.019	−0.057 *	0.164 ***	0.215 ***	0.061 *
Number of psychosomatic complaints	−0.008	0.016	−0.039	0.068 **	0.125 ***	0.179 ***
Hours of sport	0.035	0.021	0.076 **	−0.091 ***	−0.206 ***	−0.187 ***
General health state	0.026	−0.010	−0.046	−0.089 ***	−0.171 ***	−0.128 ***
Parental help with school problems	0.032	0.017	0.118 ***	0.086 **	0.053 *	−0.016
School stress	0.032	0.031	0.032	0.135 ***	0.176 ***	0.160 ***

* *p* < 0.05; ** *p* < 0.01; *** *p* < 0.001. Abbreviations: TBP, technology-based parenting; FAS III, Family Affluence Scale III; MSPSS, Multidimensional Scale of Perceived Social Support; SDQ, Strengths and Difficulties Questionnaire; BMI, body mass index.

**Table 3:** Distribution of daily digital media use per age group.

	6–9 Years	10–13 Years	14–17 Years
	N (%)	N (%)	N (%)
0–0.5 h	775 (35.5%)	141 (7.3%)	234 (12.9%)
1–2 h	1140 (52.2%)	842 (43.8%)	
2.5–3.5 h	225 (10.3%)	691 (36.0%)	611 (33.7%)
4 h or more	44 (2.0%)	248 (12.9%)	969 (53.4%)

**Table 4:** Marginal adjusted mean values with 95% confidence intervals (CIs) of the two-way ANOVA for hours of digital media use and use of TBP.

Outcome	0–0.5 h		1–2 h		2.5–3.5 h		4+ h		TBP Yes		TBP No	
10–13 years	mean	95% CI	mean	95% CI	mean	95% CI	mean	95% CI	mean	95% CI	mean	95% CI
SDQ total score	7.69	6.67;8.70	6.87	6.38;7.36	8.45	7.88;9.01	8.43	7.64;9.22	8.34	7.71;8.98	8.26	6.82;9.71
SDQ Hyperactivity score	2.61	2.20;3.03	2.38	2.17;2.58	2.88	2.65;3.11	2.77	2.45;3.10	2.86	2.69;3.03	2.47	2.21;2.72
SDQ conduct score	1.49	1.20;1.77	1.46	1.32;1.60	1.72	1.56;1.88	2.02	1.80;2.24	1.79	1.67;1.91	1.55	1.38;1.72
14–17 years			0–2 h		2.5–3.5 h		4+ h		TBP yes		TBP no	
Hours of sleep			8.54	8.44;8.64	8.26	8.19;8.32	7.98	7.93;8.04	8.36	8.29;8.43	8.16	8.10;8.22
BMI			20.24	19.82;20.65	19.92	19.66;20.17	20.37	20.15;20.59	20.07	19.81;20.34	20.27	20.04;20.51

Values represent marginal adjusted mean outcomes with 95% confidence intervals derived from two-way ANOVA models, including technology-based parenting (TBP; yes/no) and categories of daily digital media use as main effects and their interaction. Results are shown separately for early adolescents (10–13 years) and late adolescents (14–17 years). Digital media use categories were defined as 0–0.5 h, 1–2 h, 2.5–3.5 h, and ≥4 h per day for adolescents aged 10–13 years; for adolescents aged 14–17 years, the first two categories were aggregated to 0–2 h. Abbreviations: TBP, technology-based parenting; SDQ, Strengths and Difficulties Questionnaire; BMI, body mass index; CI, confidence interval.

## Data Availability

The data presented in this study are available from the corresponding author upon reasonable request.

## Abbreviations

The following abbreviations are used in this manuscript:

ASTAT	Landesinstitut für Statistik der Autonomen Provinz Bozen—Südtirol
BMI	Body Mass Index
CASMIN	Comparative Analysis of Social Mobility in Industrial Nations
CI	Confidence Interval
df	Degrees of Freedom
FAS	Family Affluence Scale
HBSC-SCL	Health Behavior in School-aged Children Symptom Checklist
HLS-EU-Q16	European Health Literacy Scale with 16 questions
M	Mean
MSPSS	Multidimensional Scale of Perceived Social Support
n.s.	Not Significant
SD	Standard Deviation
SDQ	Strength and Difficulties Questionnaire
TBP	Technology-Based Parenting
